# Bu-Shen-Yi-Sui Capsule, an Herbal Medicine Formula, Promotes Remyelination by Modulating the Molecular Signals via Exosomes in Mice with Experimental Autoimmune Encephalomyelitis

**DOI:** 10.1155/2020/7895293

**Published:** 2020-07-22

**Authors:** Pei-Yuan Zhao, Jing Ji, Xi-Hong Liu, Hui Zhao, Bin Xue, Liang-Yun Jin, Yong-Ping Fan, Wen-Jing Zhao, Lei Wang

**Affiliations:** ^1^School of Traditional Chinese Medicine, Beijing Key Lab of TCM Collateral Disease Theory Research, Capital Medical University, Beijing 100069, China; ^2^Basic Discipline of Integrated Chinese and Western Medicine, Henan University of Chinese Medicine, Zhengzhou Henan, China; ^3^Core Facility Center, Capital Medical University, Beijing 100069, China; ^4^Beijing Tian Tan Hospital, Capital Medical University, Beijing 100070, China; ^5^Beijing Hospital of Traditional Chinese Medicine, Capital Medical University, Beijing 100010, China

## Abstract

Multiple sclerosis (MS) is a common inflammatory demyelinating disorder of the central nervous system. Bu-shen-yi-sui capsule (BSYSC) could significantly reduce the relapse rate, prevent the progression of MS, and enhance remyelination following neurological injury in experimental autoimmune encephalomyelitis (EAE), an established model of MS; however, the mechanism underlying the effect of BSYSC on remyelination has not been well elucidated. This study showed that exosomes carrying biological information are involved in the pathological process of MS and that modified exosomes can promote remyelination by modulating related proteins and microRNAs (miRs). Here, the mechanism by which BSYSC promoted remyelination via exosome-mediated molecular signals was investigated in EAE mice and oligodendrocyte progenitor cells (OPCs) in vitro. The results showed that BSYSC treatment significantly improved the body weight and clinical scores of EAE mice, alleviated inflammatory infiltration and nerve fiber injury, protected the ultrastructural integrity of the myelin sheath, and significantly increased the expression of myelin basic protein (MBP) in EAE mice. In an in vitro OPC study, BSYSC-containing serum, especially 20% BSYSC, promoted the proliferation and migration of OPCs and induced OPCs to differentiate into mature oligodendrocytes that expressed MBP. Furthermore, BSYSC treatment regulated the expression of neuropilin- (NRP-) 1 and GTX, downregulated the expression of miR-16, let-7, miR-15, miR-98, miR-486, and miR-182, and upregulated the level of miR-146 in serum exosomes of EAE mice. In conclusion, these results suggested that BSYSC has a neuroprotective effect and facilitates remyelination and that the mechanism underlying the effect of BSYSC on remyelination probably involves regulation of the NRP-1 and GTX proteins and miRs in serum exosomes, which drive promyelination.

## 1. Introduction

Multiple sclerosis (MS) is an autoimmune inflammatory demyelinating disease of the central nervous system (CNS) [1]. The etiology and pathogenesis of MS are related to heredity, the environment, viral infection, and immunity. Demyelination caused by immune inflammation is the main cause of severe neurological dysfunction in patients with MS. [2] The clinical manifestations of MS include limb weakness, fatigue, visual impairment, and abnormal sensation, and MS patients eventually develop paralysis or blindness. MS typically presents in a relapsing/remitting form, with episodes of acute demyelination and neurological dysfunction. There are more than 2.5 million MS patients worldwide aged 20-40 years old. The high recurrence rate and disability rate of MS make it a burden for families and society [3, 4].

In the treatment of MS, immunosuppressant drugs such as glucocorticoids, interferon, or fingolimod are often used to alleviate nerve injury caused by immunoregulation. However, there is still no safe and effective method for remyelination. Therefore, promoting remyelination has become an urgent and challenging problem in the treatment of demyelinating diseases, such as MS. [5] The advantage of traditional Chinese medicine (TCM) is obvious during the course of recurrence and remission MS. [6, 7] Bu-shen-yi-sui capsule (BSYSC) is a TCM formula that consists of Rehmanniae Radix (Shengdihuang), Rehmanniae Radix Praeparata (Shudihuang), Polygoni Multiflori Radix (Heshouwu), Rhei Radix et Rhizoma (Dahuang), Leonuri Herba (Yimucao), Fritillariae Thunbergii Bulbus (Zhebeimu), Hirudo (Shuizhi), Scorpio (Quanxie), Gastrodiae Rhizoma (Tianma), and Forsythiae Fructus (Lianqiao) created by Prof. Yongping Fan of Beijing Tiantan Hospital based on Liu-wei-di-huang pills (a well-known formula in the Song dynasty). BSYSC has been used to treat MS for over 15 years in China. Previous studies have confirmed that BSYSC significantly alleviates sensory abnormalities, such as limb numbness and pain, in MS patients, delays the course of disease, reduces the recurrence rate of MS, and promotes nerve recovery [8]. BSYSC also improves the neurological function score of mice with experimental autoimmune encephalomyelitis (EAE), which is an animal model of MS, reduces nerve cell damage, and promotes myelin sheath and axon regeneration [9, 10], but its mechanism remains unclear.

Oligodendrocyte precursor cells (OPCs) are recruited to injured sites, differentiate into mature myelinating oligodendrocytes (OLs) around axons, and restore nerve function after demyelination in MS. OPCs express different signaling molecules during differentiation and express myelin-specific proteins, such as myelin basic protein (MBP) and cyclic nucleotide 3-phosphohydrolase (CNPase). Although the proliferation, migration, and differentiation of OPCs occur spontaneously in MS, remyelination is often incomplete or insufficient, especially in the remission phase. Remyelination failure has been attributed to a disadvantageous microenvironment after demyelination and the disequilibrium of intrinsic and extrinsic factors, such as neuron-glial antigen (NG) 2, platelet-derived growth factor receptor alpha (PDGFR*α*), neuropilin- (NRP-) 1, and GTX, which hinder complete remyelination during the differentiation phase of OPCs [11, 12].

Recent studies have found that exosome-mediated molecular signals of OPCs play an important role in remyelination. Exosomes are membranous vesicles of nanometer size (30-100 nm in diameter) that are secreted by many types of cells, such as nerve cells and immune cells. Exosomes contain mRNAs, microRNAs (miRs), various proteins (such as Alix and HSP70), and signaling molecules (CD9 and CD63) [13]. The surfaces of exosomes from different sources have their own characteristic signaling molecules. Exosomes flow throughout the body in bodily fluids and easily penetrate the blood-brain barrier (BBB) [14]. Therefore, exosomes from different sources can be detected in blood, cerebrospinal fluid and other bodily fluids [15]. miRs play an important role in regulating gene and phenotypic modifications of receptor cells [16]. As carriers of information, exosomes play multiple roles in the regulation of the central microenvironment. For example, oligodendrocyte-derived exosomes can regulate myelin sheath formation [17] and nerve axon integrity [18], which plays an important role in stabilizing the microenvironment. Exosomes also convey disease information, such as the increase in cerebrospinal fluid exosomes in MS patients and EAE mice, and are closely related to the severity of disease [19]. Exosomes from endothelial cells promote monocyte and lymphocyte to penetration of the BBB, and a diffuse inflammatory response can cause demyelination [20]. However, modified exosomes can deliver relevant information and promote regeneration of the myelin sheath [21].

Based on previous research results, our goals are to detect the expression of NRP-1, GTX, and miRs, which are the major molecular signals that affect immune and remyelination in serum exosomes of EAE mice, and to explore the ability of BSYSC to promote remyelination by modulating molecular signals mediated by exosomes in MS to provide a scientific context for invigorating the kidney and producing marrow to treat MS.

## 2. Materials and Methods

### 2.1. Animals

C57BL/6 female mice (16-18 g) were purchased from the Experimental Animal Center of the Academy of Military Medical Sciences (SCXK (Military) 2012-0004) and were bred at the Experimental Animal Center of Capital Medical University (SCXK (Beijing) 2010-0020) under specific pathogen-free conditions. Protocols for animal experiments were approved by the Animal Experiment and Experimental Animal Welfare Committee of Capital Medical University (approval number: AEEI-2015-185).

### 2.2. Preparation of BSYSC

BSYSC consists of 10 Chinese herbs: Rehmanniae Radix (Shengdihuang); Rehmanniae Radix Praeparata (Shudihuang); Polygoni Multiflori Radix (Heshouwu); Rhei Radix et Rhizoma (Dahuang); Leonuri Herba (Yimucao); Fritillariae Thunbergii Bulbus (Zhebeimu); Hirudo (Shuizhi); Scorpio (Quanxie); Gastrodiae Rhizoma (Tianma); and Forsythiae Fructus (Lianqiao). The ratio of these herbs is 10 : 10 : 10 : 2 : 10 : 6 : 3 : 2 : 3 : 6. BSYSC was prepared by Beijing Yadong Bio-Pharmaceutical Co., Ltd. The preparation process was as follows. Except for Fritillariae Thunbergii Bulbus, which was crushed into a fine powder, the other nine herbs were immersed in distilled water for 30 min and boiled for 2 h. Inspissation of the filtered solution was performed under reduced pressure at 70°C, and then, the dried powder was uniformly mixed with Fritillariae Thunbergii Bulbus. To ensure the quality and stability of BSYSC, the characteristic chemical fingerprint and the main chemical components of BSYSC were identified using ultraperformance liquid chromatography quadrupole time-of-flight mass spectrometry (UPLC-QTOF-MS) [9, 22]. The same batch of BSYSC was used throughout our study.

### 2.3. EAE Induction

Mice were randomly divided into four groups: [1] normal control (NC); [2] EAE; [3] EAE + prednisone acetate (PA, Zhejiang Xianju Pharmaceutical Co., Ltd, Zhejiang, China) 6 mg/kg; and [4] EAE + BSYSC 3.02 g/kg. EAE mice were induced by subcutaneous injection of 0.2 mL emulsion containing 50 *μ*g myelin oligodendrocyte glycoprotein (MOG) _35–55_ in 100 *μ*L of complete Freund's adjuvant (Sigma-Aldrich, St. Louis, MO, USA) supplemented with 0.3 mg of mycobacterium tuberculosis (BD Biosciences, San Diego, CA, USA) at 0 and 7 days postinduction (dpi). Then, mice were intraperitoneally (i.p.) injected with 500 ng of pertussis toxin (Sigma-Aldrich, St. Louis, MO, USA) on 0 and 2 dpi. EAE mice were treated with distilled water, 6 mg/kg PA, or 3.02 g/kg BSYSC by oral gavage once a day for 40 days. BSYSC treatment was started at 0 dpi in preventive mode. The previous experimental results showed that this kind of continuous administration of BSYSC was safe and effective; on the basis of studies, the mechanism underlying the effect of BSYSC was explored with the same model and administration in the study. In addition, the mice with EAE were received different doses of BSYSC. BSYSC at 3.02 g/kg produced the best effect on regulating immunity and promoting nerve regeneration [9, 10, 15], so we chose this dose for preventive administration.

### 2.4. Body Weight and Clinical Score in Mice

From the first day of modeling, the daily progression of the body weight and clinical scores can reflect the progression of the disease. The clinical scores used the following criteria [23]. For the tail, 0 indicated no symptoms, 1 indicated a half-paralyzed tail, and 2 indicated a fully paralyzed tail. For each of the hind or forelimbs, 0 indicated no symptoms, 1 indicated weak or altered gait, 2 indicated paresis, and 3 indicated a fully paralyzed limb. The total score was obtained by summing these scores; asymptomatic was indicated by a score of 0, a fully paralyzed quadriplegic animal was indicated by a score of 14, and mortality was indicated by a score of 15.

### 2.5. H&E Staining

Mice were sacrificed at 20 or 40 dpi. Mice were anesthetized via 3.5% hydrated chloral anesthetization, and physiological saline was replaced with a slow drip of 4% polyformaldehyde for 30 min after rapid perfusion via the heart. Then, the mouse brain and spinal cord (SC) were carefully removed and placed in 4% polyformaldehyde. To evaluate inflammation and demyelination, paraffin-embedded sections (4 *μ*m) from the mouse brain and SC were stained with hematoxylin and eosin (H&E). Five nonoverlapping fields of view were randomly selected in each slice; the integral optical density was determined using the NIS-Elements BR 3.0 system for semiquantitative analysis, and the inflammation scores were evaluated.

### 2.6. Transmission Electron Microscopy (TEM) Analysis

For ultramicrostructural analysis of myelin, mice were perfused for at least 30 min with 0.01 M PBS containing 4% formaldehyde and 2% dialdehyde, and the tissue was fixed with 2.5% glutaraldehyde for 2 h. The slices were cut and fixed with 2% osmium, rinsed, dehydrated, and embedded. Ultrathin slices were evaluated using an electron microscope (JEM-2100, JEOL, Tokyo, Japan). The g-ratio [24] was calculated based on the use of Image-Pro Plus 6.0 (Media Cybernetics, Silver Spring, MD, USA) using at least 50-100 randomly selected myelin axons.

### 2.7. Immunofluorescence Staining of the Brain and SC of Mice

After the mice were sacrificed at 20 or 40 dpi, paraffin-embedded tissue sections were cut into 5 *μ*m pieces. The slices used for immunofluorescence staining were incubated in medium containing 10% normal serum. The slices were incubated with an MBP antibody (Abcam, Cambridge, MA, USA, 1 : 400) at 4°C for 2 days. After incubation with a goat anti-rabbit antibody at 37°C for 2 h, the slices were counterstained with 4,6-diamino-2-phenyl indole (DAPI, Southern Biotech, AL, USA). A representative image was obtained using the NIS-Elements BR 3.0 system (Nikon, Tokyo, Japan), and an integrated optical density (IOD) value was calculated for further data analysis.

### 2.8. Isolation of Exosomes from the Serum of Mice

Blood samples were collected from mice via cardiac blood collection at 20 and 40 dpi. The sample was centrifuged at 3000 × g. The serum was collected. Then, only the prefiltered serum was used, excluding particles larger than 0.8 *μ*m. The sample and buffer were added to the column and centrifuged. These steps followed the instructions of the Qiagen Kit. Finally, the liquid was centrifuged at 5000 × g for 5 min to acquire exosomes.

### 2.9. Identification of Exosomes Isolated from the Serum of Mice

#### 2.9.1. Morphological Observation of Exosomes by TEM

Exosomes were diluted and mixed using the same volume of distilled water, dropped on a copper mesh, set aside for 10 min, restained with a 2% phosphoric acid solution for 30 s, rinsed with distilled water 3 times, and dried. The exosome morphology was observed using TEM.

#### 2.9.2. Atomic Force Microscopy (AFM)

The surface features of the exosomes were observed by AFM imaging in 3 dimensions. To fix exosomes on a desiccated glass cover slip, 10 *μ*L of a 10^−3^ diluted exosomes culture was gently pipetted onto a cover slip, the samples were thoroughly rinsed with 5 mL of distilled water, and the specimen was dried with filter paper for 12 h. Then, the samples were observed via preliminary scanning several times using an AFM FPAPE-AFM-A100 (Applied Physics & Electronics, Inc., A.P. E Research, Mexico, USA). Noncontact mode images were taken with a silicon-etched Ultrasharp™ probe tip (MikroMasch, USA) with a 10 nm radius and a spring constant of 40 nm in tapping mode in air at room temperature to measure the height and deflection of the specimens. AFM images were processed with the scanning probe microscope (SPM) lab 4.0 Software and analyzed with the WSxM SPM software 2.0. For the purpose of comparison, the root mean square (RMS) roughness was chosen to investigate the topographic alterations of gutta-percha and Resilon cones.

#### 2.9.3. Dynamic Light Scattering (DLS) Method

Exosomes sizes were measured by DLS using a Nano ZS90 system (Malvern Instruments, Malvern, UK) [25]. Ten microliters of exosomes were diluted in 990 *μ*L of PBS (1 : 100) and then mixed to provide a homogeneous solution; the solution was then transferred to a disposable cuvette for size measurements. The particle size distribution was measured using a Nano ZS90 light scattering analyzer at 25°C, and 10 spectra were recorded. The measurement was repeated 3 times. Data were acquired and analyzed using dispersion technology software (V7.01).

#### 2.9.4. Western Blot Analysis

Total proteins from exosomes were extracted by lysis in the presence of protease inhibitors, and 24 *μ*g of each sample was analyzed by sodium dodecyl sulfate polyacrylamide gel electrophoresis (SDS-PAGE). After electrophoresis, proteins were transferred to PVDF membranes (Merck MilliporeSigma, Billerica, MA, USA). The membranes were blocked using 5% milk protein in TBST, and the transfer membranes were incubated overnight at 4°C with antibodies against Alix (1 : 1000, Abcam, Cambridge, MA, UK), CD9 (1 : 1000, Abcam, Cambridge, MA, UK), NRP-1 (1 : 200, Abcam, Cambridge, MA, UK), GTX (1 : 5000, Abcam, Cambridge, MA, UK), or *β*-actin (1 : 20000, GeneTex, TX, USA). After incubation with the corresponding secondary antibodies, the membranes were washed 3 times with TBST. The final detection was performed with enhanced chemiluminescence using the Fusion FX6 XT System. The optical density value of each band was quantified using ImageJ and normalized to the corresponding *β*-actin level.

### 2.10. High-Throughput Sequencing of miRs from Serum Exosomes of Mice

High-throughput sequencing of miRs from serum exosomes of mice was performed by Annoroad Gene Technology (Beijing) Co., Ltd. In short, total RNA extracted from exosomes was normalized via agaric electrophoresis and an ultraviolet spectrophotometer. A total of 18-30-nt RNA fragments were collected through gum separation, and 3′ and 5′ connectors were connected at both ends of the obtained RNA. Reverse transcription was performed using cDNA, and the RNAs were sequenced using the Illumina HiSeq platform.

#### 2.10.1. Differentially Expressed miRs

miRs expression from the mirDeep2 outputs were used for analysis. Reads per million (RPM) were calculated as follows: the read number of miRs/total clean reads mapped to genome × 1,000,000. The Pearson correlation coefficients of the miRs expression levels were used to measure the purity of the samples. Differential expression analyses were performed using the DESeq2 package of bioconductor. Differentially expressed miRs were filtered with the following thresholds: false − discovery rate (FDR) ≤ 0.05 and ∣log2 fold change (Log2 FC) | ≥1.

#### 2.10.2. Pathway and Network Analyses of miRs

The targets of differentially expressed miRs were predicted using the miRBase and GenCode prediction tools. The targets predicted by both prediction tools were considered for further analysis.

### 2.11. Measurement of miRs by Quantitative Real-Time Polymerase Chain Reaction (qRT-PCR)

The expression level of the destination miRs was confirmed by qRT-PCR using a one-step qRT-PCR kit (Toyobo, Osaka, Japan). U6 was used to normalize the miRs. The level of expression of miRs was quantified using the Bio-Rad CFX real-time system and was analyzed using the CFX management software v2.0 (Bio-Rad, Hercules, CA). Relative quantification of miRs was performed using the 2^-*ΔΔ*Ct^ method, and each sample was normalized.

### 2.12. OPCs Culture

The brain tissue was obtained from P1 Sprague-Dawley neonatal rats. Briefly, after exfoliation of the cortical tissue, the single-cell suspension was obtained by physical and chemical grinding. The cells were plated in a polylysine- (PLL-) coated culture flask, cultured in DMEM/F12 medium containing 20% FBS for 7-9 days and shaken at 250 rpm/min for 16-18 h to obtain OPCs from mixed glial cells.

### 2.13. Preparation of BSYSC-Containing Serum

Adult Sprague-Dawley rats were randomly divided into 2 groups: the NC group and the BSYSC group. BSYSC powder was dissolved in distilled water (11.7 g raw herb/kg), the mice in the NC group were treated with distilled water, and the mice in the BSYSC group received BSYSC by oral gavage twice per day for 7 days. On day 7, after gavage feeding for 2 h, the blood was collected from the abdominal aorta and then centrifuged at low temperature for 20 min to avoid hemolysis. The serum of the mice was heat-inactivated in a water bath at 56°C for 30 min, sterilized through a 0.22 *μ*m microporous membrane, and placed at -80°C for use after dispensing. The main chemical constituents in BSYSC-containing serum were determined by UPLC-MS/MS. [9, 22] The same serum containing BSYSC was used in the cultured OPCs study.

### 2.14. Cell Counting Kit- (CCK-) 8 Assay

The proliferation of OPCs was detected by the CCK-8 assay. The cells were seeded in PLL-coated 96-well plates at a density of 8000 cells per well and cultured for 12 h using OPC proliferation medium that was replaced with different concentrations of BSYSC-containing serum (5%, 10%, and 20%). After 24 h, the cells were incubated in basal medium containing 10 *μ*L of CCK-8 for 1 h, and the absorbance of each well at 450 nm was measured with a microplate reader.

### 2.15. Immunofluorescence Staining

To characterize cells according to immunofluorescence, the cells were briefly washed with PBS, fixed in 4% paraformaldehyde for 15 min, and then washed 3 times with PBS for 5 min each. OPCs were treated with 0.3% Triton-100 for 10 min and blocked with 5% BSA for 30 min. Then, OPCs were incubated with a rabbit anti-MBP primary antibody at 4°C overnight. After washing with PBS, the OPCs were incubated with a secondary antibody for 1 h at room temperature. To visualize nuclei, DAPI staining was performed. Images were captured using a fluorescence microscope.

### 2.16. Boyden Chamber Assay

A polylysine solution was added to the lower chamber to infiltrate both sides of the Transwell membrane. The chamber was placed at 37°C for 30 min, rinsed twice with ddH_2_O, and dried under sterile conditions for use. The cells were digested, the culture was discarded by centrifugation, the medium was resuspended, and the Transwell membrane was wetted with 100 *μ*L of basal medium. OPCs were seeded into the upper chamber (200 *μ*L) at 6 − 8 × 10^4^ cells per Transwell, and 600 *μ*L of OPCs proliferation medium was added to the lower chamber. After the OPCs attached, the upper medium was changed to contain different concentrations of BSYSC-containing serum (5%, 10%, and 20%). After the cells migrated for 24 h, the medium was aspirated, fixed with 4% paraformaldehyde for 15 min at room temperature, and stained with 0.1% crystal violet for 15 min. Finally, the unmigrated cells on the upper side of the chamber were wiped with a cotton swab under a microscope.

### 2.17. Statistical Analysis

Data are presented as the mean ± SEM. One-way ANOVA for repeated measurements was used to calculate statistical significance. Differences for which *P* < 0.05 were considered statistically significant.

## 3. Result

### 3.1. BSYSC Treatment Improves the Body Weight and Clinical Scores of EAE Mice

**Figure 1:**
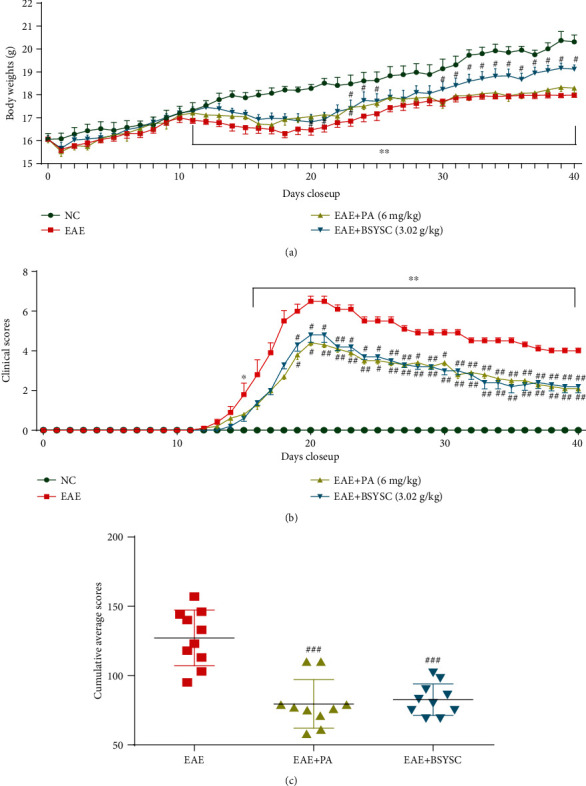
BSYSC treatment improves the body weight and clinical scores of EAE mice. (a) Changes in body weight for NC (*n* = 10), EAE (*n* = 10), PA (*n* = 10), and BSYSC (*n* = 10) mice. (b) The clinical scores of different groups were assessed. (c) The cumulative average clinical scores of different groups were evaluated. Data are expressed as the mean ± SEM. ∗∗*P* < 0.01 vs. the NC group and ^#^*P* < 0.05, ^##^*P* < 0.01 vs. the EAE group.

The body weight of EAE mice was significantly reduced compared with that of NC mice at 11-40 dpi (*P* < 0.01). Compared with that of EAE mice, the body weight of PA-treated mice was markedly elevated at only 20, 21, and 23 dpi (*P* < 0.05), while the weight of BSYSC-treated mice was increased at 23-25 and 30-40 dpi (*P* < 0.05, Figure 1(a)).

The clinical scores of mice with EAE were evaluated at 12 dpi, and the scores peaked at 6.5 points at 20 dpi. The scores gradually decreased; however, at 16 dpi, compared with the NC group, the difference in scores was statistically significant (*P* < 0.05 or *P* < 0.01). The overall trend of PA- and BSYSC-treated mice was consistent with that of EAE mice, but the scores of both PA- and BSYSC-treated mice were significantly reduced at 19-40 dpi compared with those of EAE mice (*P* < 0.05 or *P* < 0.01, Figure 1(b)). The cumulative average scores of PA- and BSYSC-treated mice were also significantly decreased compared with those of EAE mice (*P* < 0.05 or *P* < 0.01, Figure 1(c)).

### 3.2. BSYSC Reduces Inflammatory Infiltration of Brain Tissue and the SC in EAE Mice

**Figure 2:**
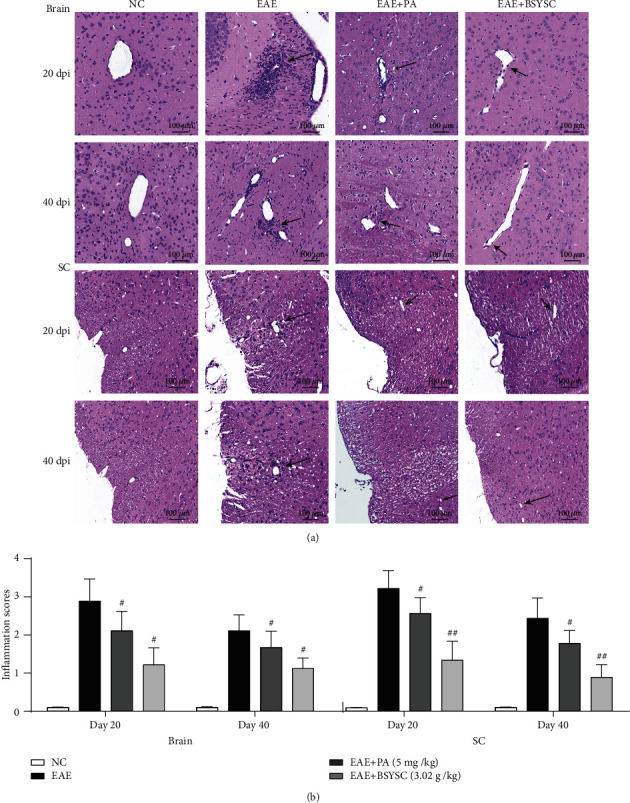
BSYSC reduces inflammatory infiltration in the brain and SC of EAE mice. (a) Inflammatory infiltrates of the brain and SC of mice at 20 and 40 dpi were observed by H&E staining. The pictures were taken at ×200 magnification. (b) The inflammatory scores were assessed. Arrows indicate inflammatory cells. Data are expressed as the mean ± SEM, ^#^*P* < 0.05, ^##^*P* < 0.01 vs. the EAE group.

The brain and SC in NC, EAE, PA-, and BSYSC-treated mice were analyzed for histological evidence of inflammation at 20 and 40 dpi. Intact structures with a clearly visible cytoplasm were found in the brain and SC of NC mice. A large number of infiltrated lymphocytes and typical sleeve-like changes around blood vessels were found in the brain and SC of EAE mice. However, the inflammatory infiltrates were improved in PA- and BSYSC-treated mice (Figure 2(a)), and a significant decrease in the inflammatory scores was found in PA- and BSYSC-treated mice compared with EAE mice (*P* < 0.05 or *P* < 0.01, Figure 2(b)).

### 3.3. BSYSC Protects the Ultrastructural Integrity of Myelin in EAE Mice

**Figure 3:**
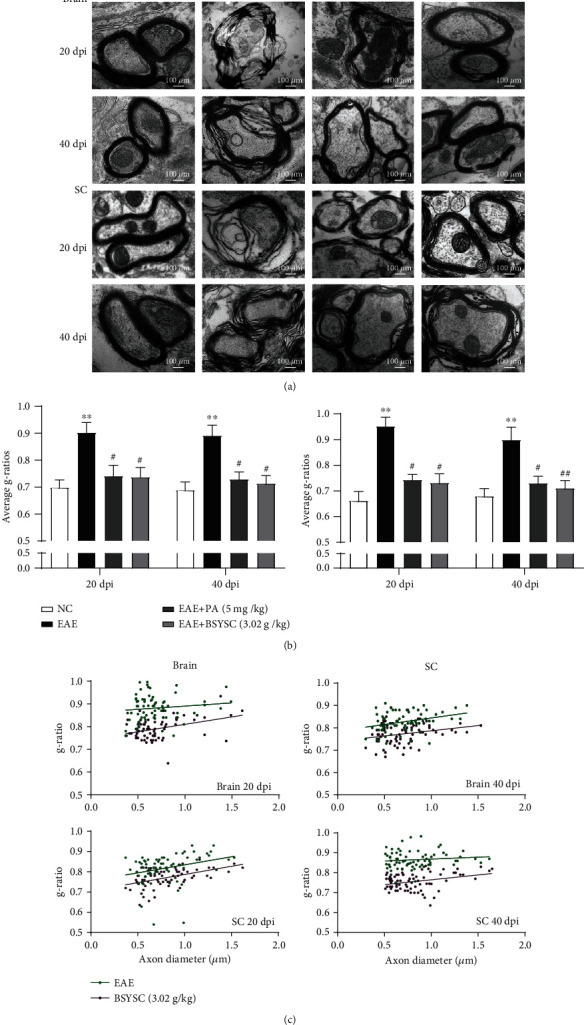
Treatment with BSYSC improves myelin deficits in EAE mice. (a) Observation of the ultrastructure of the myelin sheath in the brain and SC of mice at 20 and 40 dpi by TEM. The pictures were taken at ×20,000 magnification. (b) The ratio of the diameter of the axon to the diameter of the fiber (g-ratio) was measured to further quantify the extent of myelination. Data are expressed as the mean ± SEM (*n* = 4 at each time point), ∗∗*P* < 0.01 vs. the NC group, ^##^*P* < 0.01 vs. the EAE group. (c) Scatter plots of the axonal diameter and g-ratio in the brain and SC at 20 and 40 dpi.

As shown by TEM, the structure of the myelin lamina layer was clear and dense, and no demyelination was observed in the NC group. The structure of the myelin lamina layer in the EAE group was loose with a decreased lamina density, and axon atrophy was reduced compared with that in the NC group at 20 and 40 dpi. The structure and density of the lamella in PA- and BSYSC-treated mice were improved to varying degrees (Figure 3(a)).

The g-ratio is the ratio of the diameter of the axon to the diameter of the axon plus the surrounding myelin in TEM observations. A higher g-ratio of the axon and myelin units indicates thinner myelin sheaths and incomplete remyelination. A significant increase in the mean g-ratio was found in EAE mice compared with NC mice at 20 and 40 dpi (*P* < 0.01); remyelinated axons were sparsely identified as thinly myelinated axons and unmyelinated axons, which were accompanied by a large number of demyelinated axons. The g-ratio in PA- and BSYSC-treated mice was significantly reduced compared with that in EAE mice (*P* < 0.05 or *P* < 0.01, Figures 3(b) and 3(c)), which exhibited a higher percentage of myelinated fibers, a lower g-ratio, and a greater myelin sheath thickness after treatment with BSYSC.

### 3.4. BSYSC Upregulates the Expression of MBP in the Brain and SC of EAE Mice

**Figure 4:**
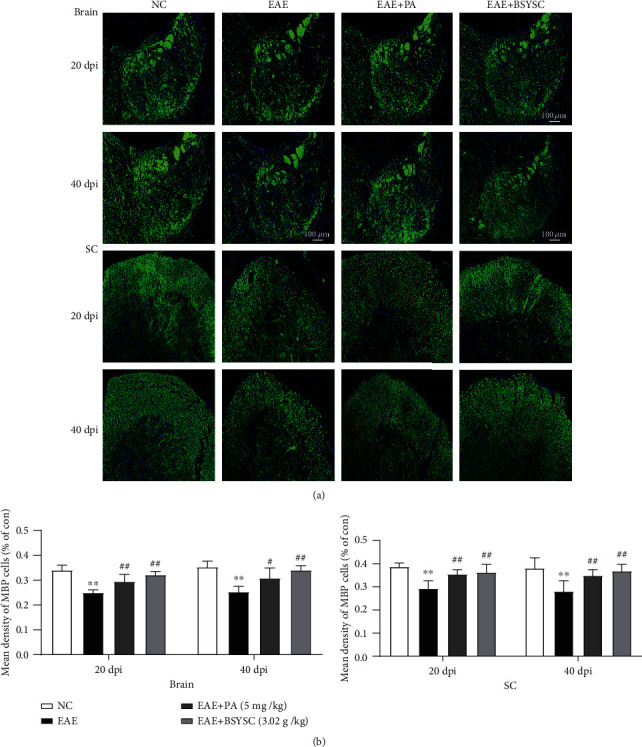
BSYSC treatment regulates MBP expression in EAE mice. Representative mice with MBP expression in the brain and SC from each experimental group were subjected to fluorescence microscopy and quantitative analysis at 20 and 40 dpi. Data are expressed as the mean ± SEM (*n* = 4 per group at each time point), ∗∗*P* < 0.01 vs. the NC group, ^#^*P* < 0.05, ^##^*P* < 0.01 vs. the EAE group.

MBP is a marker of oligodendrocyte maturation, and the expression of MBP in the brain, and SC of the mice in each group was detected by immunofluorescence (Figure 4(a)). The results showed that MBP levels in the brain and SC of EAE mice were significantly lower than those in the brain and SC of the NC group at 20 and 40 dpi (*P* < 0.01). In contrast, MBP was increased in PA- and BSYSC-treated mice compared with EAE mice (*P* < 0.01, Figure 4(b)).

### 3.5. Isolation and Characterization of Serum Exosomes Derived from Mice

**Figure 5:**
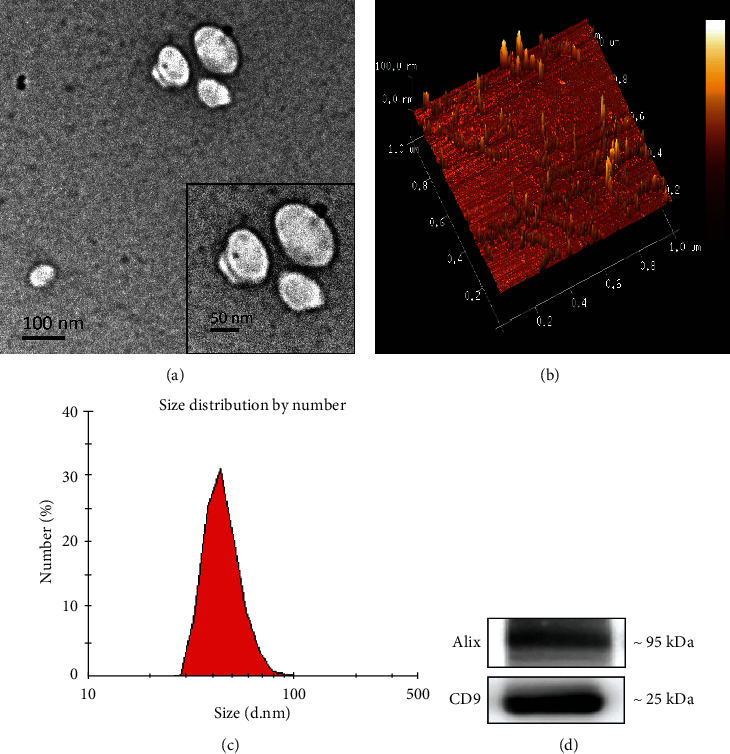
Characterization of serum exosomes. (a) TEM image depicting the spherical morphology of isolated exosomes; AMF (b) and DLS (c) illustrate the size distribution of the isolated exosomes. (d) Western blot analysis showing the presence of Alix and CD9 in exosomes isolated from mouse serum.

For the purpose of extracting miRs from serum exosomes in mice, serum was collected at 40 dpi, and an exoEasy Kit (Qiagen, Germany) was used with a membrane affinity spin column. Consistent with the general characteristics of exosomes, TEM showed that the obtained particles were spherical structures with a diameter of 100 nm (Figure 5(a)). The diameter of the particles was found to be distributed from 30 to 100 nm using AFM and DLS (Figures 5(b) and 5(c)). Furthermore, the exosome marker proteins Alix and CD9 were detected by Western blot analysis, and it was confirmed that the isolated particles could express these two types of proteins (Figure 5(d)). The above results indicate that the particles obtained from mouse serum were confirmed to exhibit the main features of exosomes. Therefore, this method can be used for subsequent experiments.

### 3.6. BSYSC Regulates the Protein Expression of NRP-1 and GTX in Serum Exosomes from EAE Mice

**Figure 6:**
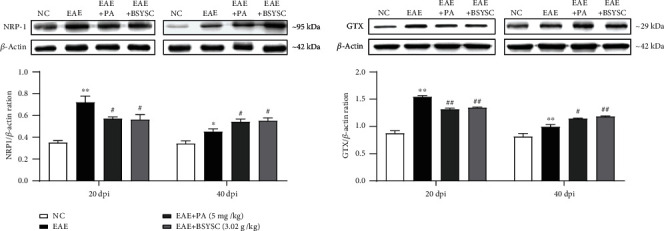
BSYSC treatment regulates the protein expression of NRP-1 and GTX in serum exosomes of EAE mice. Representative images of Western blot and quantitative analyses of NRP-1 and GTX in the serum exosomes of mice at 20 and 40 dpi. *β*-Actin was used as the internal standard. Data are expressed as the mean ± SEM. ∗*P* < 0.05, ∗∗*P* < 0.01 vs. the NC group and ^#^*P* < 0.05, ^##^*P* < 0.01 vs. the EAE group.

Proteins related to myelin expression of NRP-1 and GTX in serum exosomes of mice were detected by Western blot analysis. At 20 dpi, compared with NC mice, EAE mice showed a significant increase in the protein expression of NRP-1 and GTX in in the brain and SC (*P* < 0.01). However, the above proteins were decreased in EAE mice treated with BSYSC compared with EAE mice (*P* < 0.05, *P* < 0.01). At 40 dpi, NRP-1 and GTX were also increased in EAE mice compared with NC mice (*P* < 0.05, *P* < 0.01). NRP-1 and GTX in BSYSC mice compared with mice from the EAE group was significant (*P* < 0.05, *P* < 0.01, Figure 6).

### 3.7. Sequencing of miRs in Serum Exosomes from Mice

**Figure 7:**
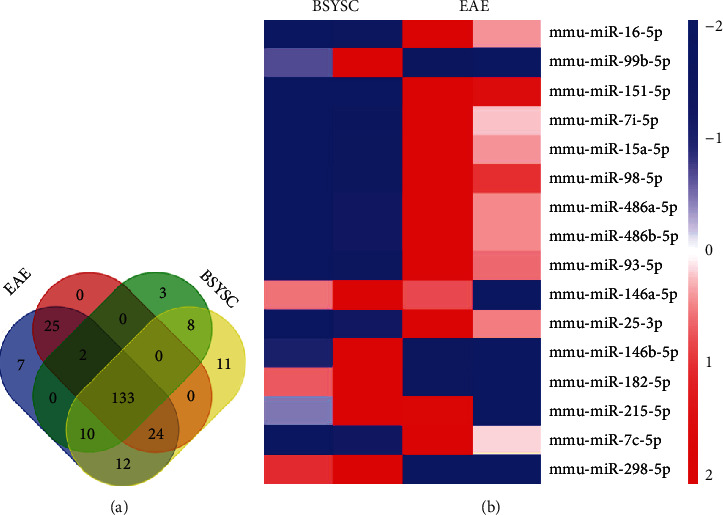
Heat map from deep-sequencing depicting the most significant differentially expressed miRs from exosomes from the EAE and BSYSC groups. (a) Venn diagram of the expressed miRNAs in each group. (b) Heatmap of 16 differentially expressed miRs; red, upregulated miRs; blue, downregulated miRs.

To characterize miRs in exosomes, the Illumina HiSeq high-throughput platform was utilized for miR sequencing. Initially, 20,840,494 and 21,631,682 raw reads were generated in the EAE group, and 20,851,101 and 21,522,865 raw reads were generated in the BSYSC group. In the EAE group, 18,125,932 and 19,568,611 clean reads were generated, while 18,344,439 and 19,364,818 clean reads were produced in the BSYSC group after trimming the low-quality read and adapter sequences. Using miRBase 21.0 as a reference, 213 and 184 known miRs were identified in the EAE group, and 156 and 198 known miRs were identified in the BSYSC group. Furthermore, 133 miRs were identified in both groups (Figure 7).

### 3.8. Changes in the Expression of 16 miRs in Serum Exosomes from Mice in the EAE and BSYSC Groups

**Figure 8:**
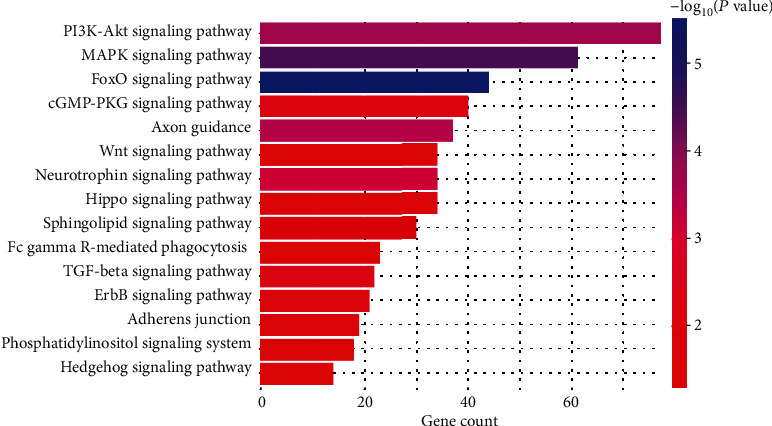
Top KEGG pathways regulated by the predicted target genes of the 16 significantly DE miRs.

Next, we identified the miRs in exosomes that differed between the EAE and BSYSC groups. Using FDR ≤ 0.05 and ∣Log2 FC | ≥1 as thresholds, 16 miRs were found to be significantly and differentially expressed (DE) between the two groups. Among them, 6 different kinds of miRs (miR-99b-5p, miR-182-5p, miR-146b-5p, miR-146b-5p, miR-215-5p, and miR-298-5p) were upregulated and 10 miRs (miR-16-5p, miR-151-5p, let-7i-5p, miR-15a-5p, miR-98-5p, miR-486a-5p, miR-486b-5p, miR-93-5p, miR-25-3p, and let-7c-5p) were downregulated in exosomes derived from mice treated with BSYSC compared to those from EAE mice (Table 1 and Figure 8).

### 3.9. Functional Pathway Analysis of miRs

**Figure 9:**
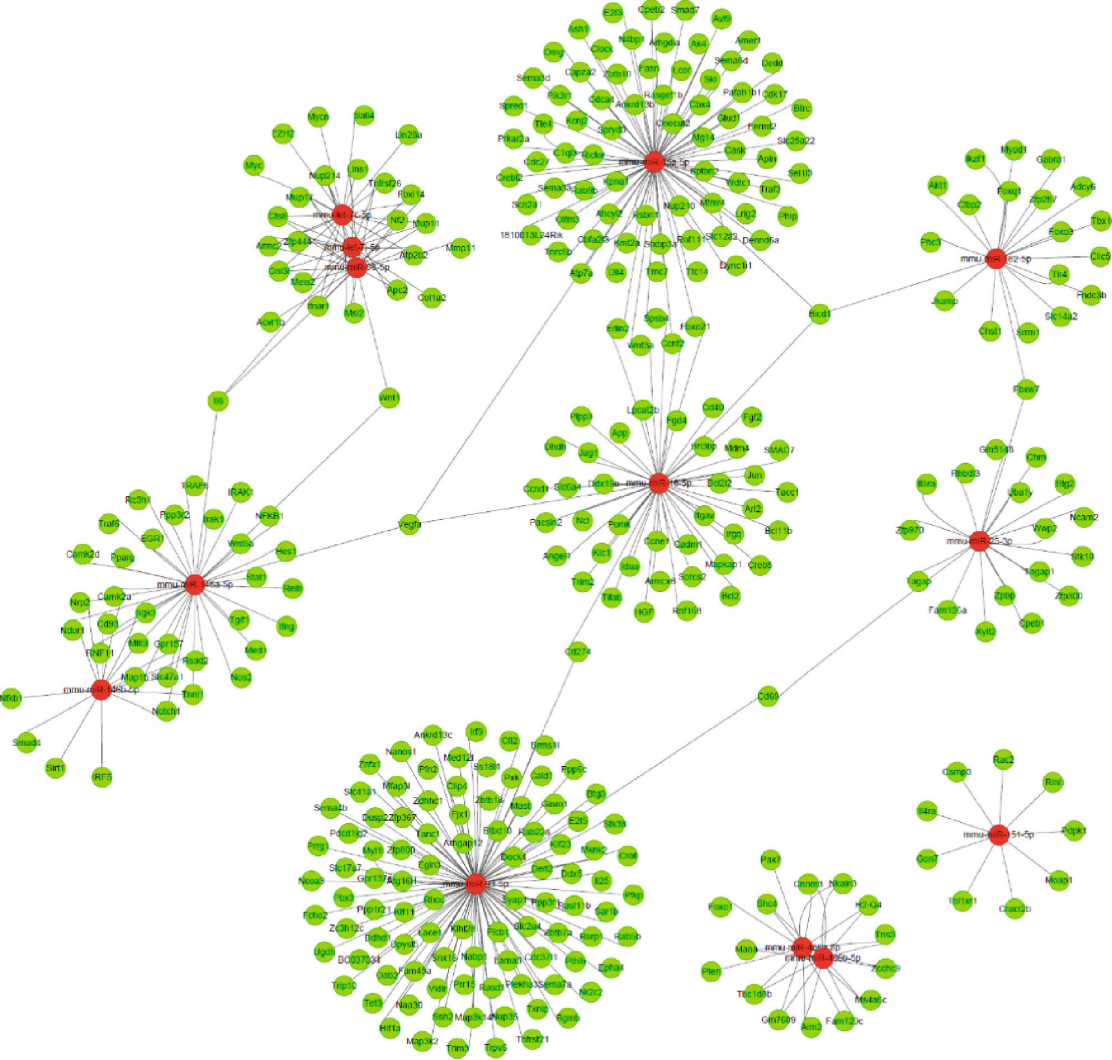
MiR-mRNA interactions of 16 DE miRs in exosomes.

To assess the potential role of exosomal miRs in the context of EAE biology and the microenvironment, the predicted functions of the DEMs were investigated using the DIANA mirPath v.3 software. Analyses were performed for the Kyoto Encyclopedia of Genes and Genomes (KEGG) pathways and GO biological processes. GO analysis identified biological processes, molecular functions, and cellular components. Ten DE miRs in exosomes were obviously associated with the regulation of neuron differentiation, cellular response to growth factor stimulus, cell migration, GTPase activity, and axonogenesis. The molecular functions influenced by the predicted target genes of the top 10 DE miRs in exosomes from serum included phospholipid binding, GTPase binding, receptor binding, protein domain-specific binding, and growth factor binding. According to the KEGG pathway analysis, DE miRs were enriched in the axon guidance and following pathways: PI3K-Akt, MAPK, FoxO, cGMP-PKG, and Wnt signaling pathways (Figure 9).

### 3.10. BSYSC Regulates the Expression of 7 miRs in Serum Exosomes from Mice with EAE

**Figure 10:**
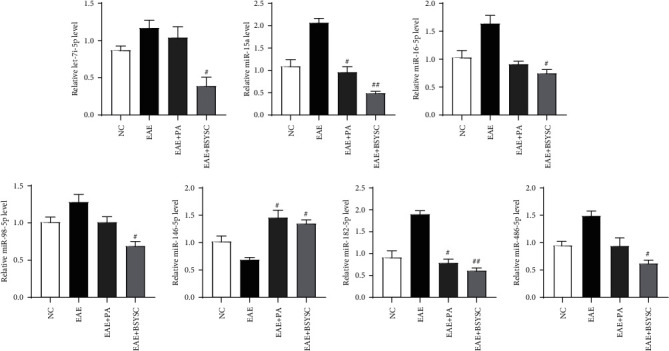
Validation of differentially expressed miRs. The relative expression of mature miRs was calculated using the 2^-*ΔΔ*Ct^ method and normalized against snoRNA202. Error bars represent the SEM. ^#^*P* < 0.05, ^##^*P* < 0.01 vs. the EAE group.

Sixteen DE miRs were verified by qRT-PCR. Seven of these miRs were consistent with the sequencing results. Sixteen genes were also identified by stem-loop qPCR, and the direction of the fold change detected was similar to the results of the microRNA-Seq analysis (Figure 10). These findings validate the miR-Seq data.

### 3.11. BSYSC-Containing Serum Promotes Proliferation, Migration, and Differentiation of OPCs In Vitro

**Figure 11:**
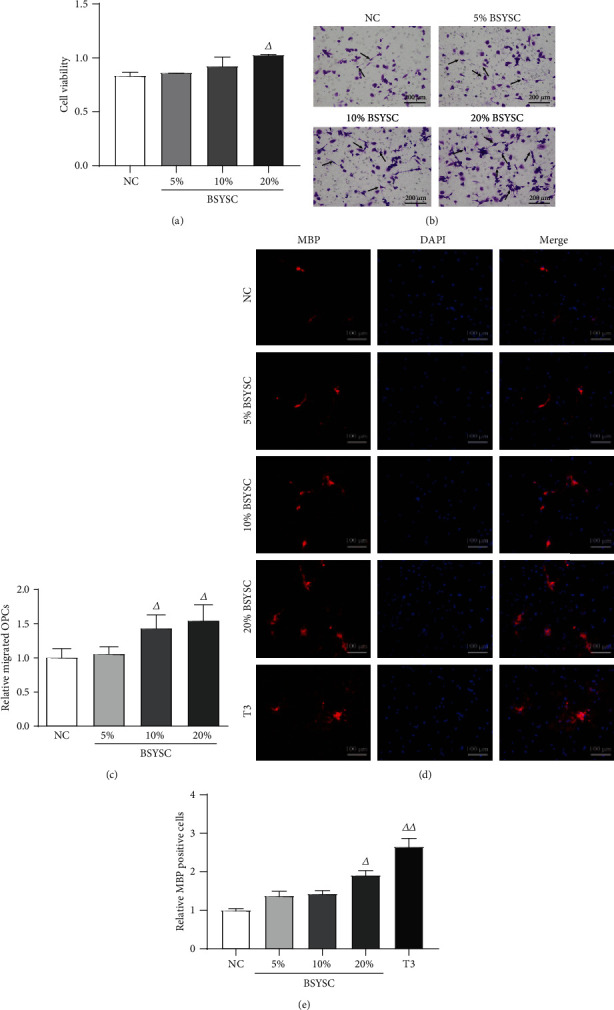
BSYSC-containing serum promotes proliferation, migration, and differentiation of OPCs in vitro. (a) The proliferation of OPCs was measured by the CCK-8 assay. (b, c) The migration of OPCs was evaluated using the Boyden chambers. Scale bar: 200 *μ*m. (d, e) The differentiation of OPCs into oligodendrocytes was determined by immunofluorescence staining showing MBP-positive cells, and the nucleus counterstained with DAPI. Scale bar: 100 *μ*m. ^△^*P* < 0.05, ^△△^*P* < 0.01 vs. the NC group.

The original OPCs were used in an in vitro culture to observe the effects of BSYSC-containing serum. The proliferation capacity of OPCs was assayed by the CCK-8 method, and the results showed that the viability of the cells in 20% BSYSC-containing serum was significantly increased compared with that of the NC group (*P* < 0.05, Figure 11(a)). The migration of OPCs was evaluated in the Boyden chambers, and OPC migration was increased in the 10% and 20% BSYSC-containing serum groups compared with that in the NC group (*P* < 0.05, *P* < 0.01, Figures 11(b) and 11(c)). The immunofluorescence assay showed that MBP expression was upregulated in 20% BSYSC-containing serum and T3 (*P* < 0.05, *P* < 0.01, Figures 11(d) and 11(e)).

## 4. Discussion

### 4.1. Neuroprotective Effect of BSYSC against MS/EAE

Although progress has been made in research on therapeutic strategies to promote remyelination in MS in recent years, effective medicines still need to be developed to treat this disease. BSYSC can significantly reduce the TCM symptom score and the disability grade score of MS patients, decrease the relapse rate, and improve quality of life. In this study, the successful establishment of the EAE model was demonstrated by weight, clinical score, H&E staining, and TEM results. The changes in the mice revealed that the weight of the mice decreased significantly after modeling compared with that of NC group, and the clinical score increased significantly; the highest score was 6.5. On H&E staining, the EAE mice showed inflammatory cell infiltration around the cerebral white matter blood vessels, obvious nuclear fixation, and obvious “cuff-like” changes, while the myelin plate structure of EAE mice was loose. TEM revealed a loose myelin plate structure in EAE mice, which showed plate separation, reduced ring density, axonal atrophy, and myelin deficiency, which confirmed the successful establishment of the EAE model. With BSYSC treatment, the mice lost less weight and regained weight more rapidly in the later stages, and their clinical scores were also reduced. H&E staining showed that the mice had inflammatory cell infiltration and partial nuclear sequestration around the white matter vessels in the brain after BSYSC treatment, which was lower than that of the EAE group. TEM revealed that mice treated with BSYSC had different degrees of loose plate structure and decreased ring density, but all features were less prominent than that in the EAE group, and axonal atrophy was less severe than that in the EAE group. This suggests that BSYSC can improve the clinical performance of EAE mice, reduce perivascular inflammatory infiltration, and promote myelin regeneration. These results are consistent with our previous studies [22, 26].

### 4.2. Ability of BSYSC to Promote Remyelination

Recent studies have shown that a large number of oligodendrocyte precursors are recruited to demyelinating lesions of MS [27], but remyelination still ends in failure, especially in the remission phase of MS. The main reason for remyelination failure is that OPCs cannot effectively differentiate into mature oligodendrocytes [28], which produce myelin. In this study, BSYSC significantly increased the expression of MBP, a marker of OPCs differentiation and maturation. In vitro, BSYSC-containing serum also increased the proliferation, migration, and differentiation of OPCs, suggesting that BSYSC promotes remyelination. In previous in vivo and in vitro studies, BSYSC has been shown to be able to upregulate myelin-related molecules, such as Olig1, Olig2/Brdu, PDGFR*α*, and CNPase in the brain tissue of EAE mice [26, 29]. Catalpol, the active ingredient of Rehmanniae Radix (Dihuang) in BSYSC, regulates the transcription factors Olig1 and Olig2 in OPCs [29, 30]. The above findings indicate that BSYSC may promote remyelination via differentiation and maturation of OPCs.

### 4.3. Mechanism Underlying the Effect of BSYSC on Remyelination

#### 4.3.1. Regulation of the NRP-1 and GTX Proteins by BSYSC in Exosomes of Mice

NRP-1, a member of the axonal glycoprotein neuropilin family, has been studied mostly with regard to its role in guiding the growth of axons and remyelination as a receptor of semaphoring 3A and vascular endothelial growth factor A (VEGF-A) [31, 32]. Further studies found that NRP-1 has been characterized in different cellular phenotypes including vascular endothelial cell, microglia, and regulatory T-cell populations [33]. Endothelial NRP-1 was upregulated in human acute MS lesions, knockout of endothelial NRP1 modulated inflammatory responses in mouse EAE model [34]. But other study of EAE found that NRP-1 related with Treg cells attenuated autoreactivity to improve the condition of EAE mice [35] and NRP-1 in microglia contributed to microglial signaling and polarization to the anti-inflammatory M2 phenotype in favor of remyelination [36]. Thus, NRP-1 plays multifaceted role in the immune system. The transcription factor GTX, an endogenous protein, affects the directional differentiation of neural stem cells, causing differentiated neural stem cells develop into oligodendrocytes [37]. GTX has multiple binding sites in the promoter sequence of protein lipid protein (PLP) and MBP and participates in the differentiation of oligodendrocytes from OPCs to OLs, which ultimately affects the formation of the myelin sheath [38, 39].

Our studies showed that NRP-1 and GTX proteins in exosomes of EAE mice are highly expressed in the acute inflammatory phase of in EAE mice, which is probably caused by severe inflammatory stimulation [40], while BSYSC downregulated the expression of the above factors thereby indicating its effects of regulating the immune and improving the inflammatory response in EAE mice. Indeed, BSYSC mediated the regulation of Th17/Treg cells in EAE mice [9]. In the remission phase, on the contrary, BSYSC upregulated NRP-1 and GTX, and the increase in NRP-1 and GTX levels contributed to remyelination in mice with EAE. Thus, BSYSC plays dual roles in both regulating immune and promoting remyelination.

#### 4.3.2. Regulation of miRs in Exosomes from Mouse Serum by BSYSC

Sixteen DE miRs in serum exosomes from mice were screened by high-throughput sequencing [41]. Remarkably, BSYSC mainly downregulated the expression of 6 miRs, namely, miR-16, let-7, miR-15, miR-98, miR-486, and miR-182, and upregulated the expression of miR-146 by qRT-PCR analysis. This study found that one of the target genes of miR-16 is brain-derived neurotrophic factor (BDNF) and that the upregulation of miR-16 was accompanied by the downregulation of BDNF in the depression model of rats. BDNF is a potent neurotrophic factor that promotes the growth and survival of neurons [42]. BDNF specifically binds to the TrkB receptor [43], which is expressed in both OPCs and oligodendrocytes. BDNF increases the number of oligodendrocytes in the basal forebrain, and BDNF deficiency in mice is associated with a decrease in the myelin sheath protein in spinal cord oligodendrocytes and the optic nerve in the early stage of development. Injection of BDNF-transfected bone marrow stem cells reduces demyelination and facilitates remyelination, thus decreasing the severity of clinical EAE symptoms. Previous studies have found that BSYSC can upregulate the expression of BDNF and TrkB protein and mRNA in the brain and spinal cord of EAE mice [22]. Let-7 is one of the most abundant miRs expressed in the brain. The functions of Let-7 include stem cell differentiation and neurogenesis regulation [44]. Let-7 is an effective agonist of Toll-like receptor (TLR) in macrophages and microglia [45, 46]. In the MOG-induced EAE model, the level of expression of the TLR7 gene is very high [47]. After application of the TLR signal inhibitor, the EAE neurological function score decreased [48]. MiR-15a was negatively correlated with B-cell lymphoma- (Bcl-) 2 [49]. After transfection of miR-15a, there was no expression of Bcl-2, and apoptosis was induced. However, in transgenic mice overexpressing Bcl-2, the inflammatory infiltration, demyelination, and axonal injury associated with EAE were significantly decreased [50]. Bcl-2 could protect neurons from apoptosis and reduce ROS-induced injury [51]. The target genes of miR-98 include insulin-like growth factor- (IGF-) 1. Overexpression of miR-98 can significantly reduce the level of endogenous IGF-1, while IGF-1 can stimulate the proliferation of human and mouse Treg cells in vitro [52]. IGF-1 treatment promotes myelin regeneration in the acute phase of EAE, but the effect is not obvious in the remission phase [53, 54]. It is speculated that the main role of IGF-1 is in the immune system, which may delay the entry of inflammatory cells into the CNS during the acute phase of MS. [55] MiR-486 was increased in spinal cord injury (SCI), and its expression was detected in motor neurons by in situ hybridization. MiR-486 was injected into the spinal cord of unimpaired mice to produce SCI-like conditions [56]. Inhibition of miR-486 can reduce ROS-mediated cell death, thereby increasing neuronal survival and leading to functional recovery. Two direct targets of miR-182 are fibroblast growth factor (FGF) 9 and neurotrimin (NTM) [57]. miR-182 can downregulate the expression of FGF 9 and NTM to inhibit the proliferation and migration of the Schwann cells [58]. The role of miR-182 in CNS demyelinating diseases remains to be studied. In addition, miR-146a is a congenital immune response mediator that targets the TRAF6 and IRAK1 genes [59]. Transfection of miR-146a promotes the differentiation of OPCs into oligodendrocytes, and the increase in miR-146a promotes the maturation of OPCs and the expression of MBP. However, miR-16, let-7, miR-15, miR-98, miR-486, miR-182, and miR-146 in serum exosomes of EAE mice were regulated by treatment with BSYSC, suggesting that the neuroprotective effects of BSYSC may be achieved either directly or indirectly by inhibiting inflammation to reduce nerve damage and promote remyelination.

In conclusion, the findings reported here indicate that the ability of BSYSC to promote remyelination in EAE mice may be closely related to its regulation of NRP-1, GTX, and related miRs. Whether BSYSC plays a direct or indirect role in the regulation of remyelination needs more studies for clarifications. In addition, because of few exosomes extracted from serum of mice, two biological replicates were used in the miRs sequencing, some miRs seem to differ greatly in the same group; this is the defects in this paper. In future experiments, we will increase the sample to provide more meaningful information.

## Figures and Tables

**Table 1:** Major GO categories regulated by 16 differentially expressed (DE) miRs in serum exosomes.

Category	Term	Involved in	*n*	*P*
Biological process	GO:0045664	Regulation of neuron differentiation	72	<0.001
	GO:0071363	Cellular response to growth factor stimulus	57	<0.001
	GO:0030334	Regulation of cell migration	74	<0.001
	GO:0043087	Regulation of GTPase activity	64	<0.001
	GO:0007409	Axonogenesis	45	<0.001

Molecular function	GO:0005543	Phospholipid binding	45	<0.001
	GO:0051020	GTPase binding	31	<0.001
	GO:0005102	Receptor binding	101	<0.001
	GO:0019904	Protein domain specific binding	54	<0.001
	GO:0019838	Growth factor binding	18	<0.001

Cellular component	GO:0097458	Neuron part	118	<0.001
	GO:0045202	Synapse	80	<0.001
	GO:0005856	Cytoskeleton	157	<0.001
	GO:0030054	Cell junction	103	<0.001
	GO:0030424	Axon	44	<0.001

## Data Availability

The data that support the findings of this study are available from the corresponding author upon reasonable request.

## Abbreviations

AFM: Atomic force microscopy
BBB: Blood-brain barrier
Bcl: B-cell lymphoma
BDNF: Brain-derived neurotrophic factor
BSYSC: Bu-shen-yi-sui capsule
CCK 8: Cell counting kit 8
CNPase: Cyclic nucleotide 3-phosphohydrolase
CNS: Central nervous system
DAPI: 4,6-Diamino-2-phenyl indole
DLS: Dynamic light scattering
EAE: Experimental autoimmune encephalomyelitis
FDR: False-discovery rate
FGF: Fibroblast growth factor
H&E: Hematoxylin and eosin
IGF: Insulin-like growth factors
IOD: Integrated optical density
MBP: Myelin basic protein
miRs: MicroRNAs
MOG: Myelin oligodendrocyte glycoprotein
MS: Multiple sclerosis
NC: Normal control
NG2: Neuron-glial antigen 2
NRP-1: Neuropilin-1
NTM: Neurotrimin
OLs: Oligodendrocytes
OPCs: Oligodendrocyte progenitor cells
PA: Prednisone acetate
PDGFR*α*: Platelet-derived growth factor receptor alpha
PLL: Polylysine
PLP: Protein lipid protein
qRT-PCR: Quantitative real-time polymerase chain reaction
RMS: Root mean square
RPM: Reads per million
SC: Spinal cord
SCI: Spinal cord injury
SDS-PAGE: Sodium dodecyl sulfate polyacrylamide gel electrophoresis
SPM: Scanning probe microscope
TCM: Traditional Chinese medicine
TEM: Transmission electron microscopy
TLR: Toll-like receptor.

## Supplementary Materials

Supplementary Materials Graphical Abstract.
