# Supplementary figures and images for: Bu-Shen-Yi-Sui Capsule, an Herbal Medicine Formula, Promotes Remyelination by Modulating the Molecular Signals via Exosomes in Mice with Experimental Autoimmune Encephalomyelitis
Source: Oxid Med Cell Longev. 2020 Jul 22;2020:7895293. doi: 10.1155/2020/7895293 (PMC7396036; doi:10.1155/2020/7895293)

## Graphical abstract

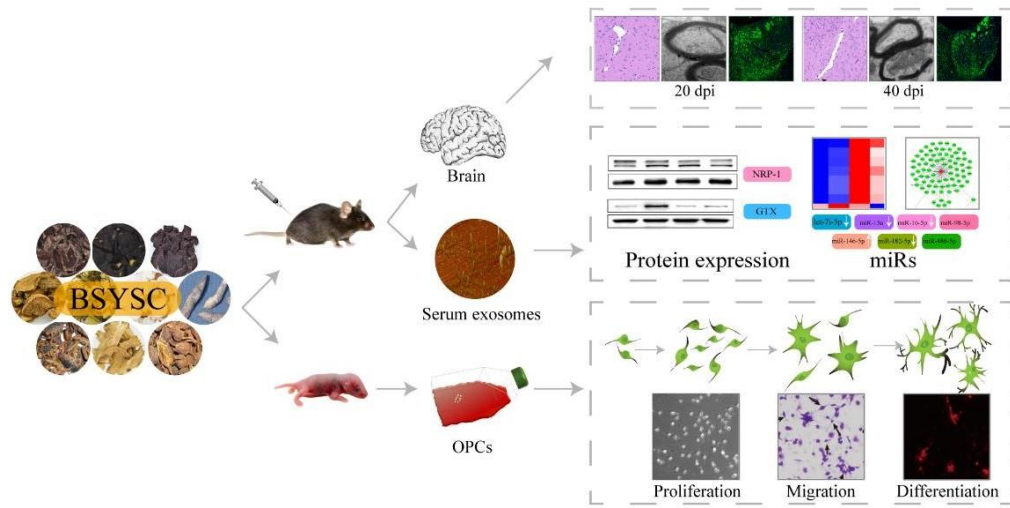

Supplement: Supplementary Materials — Graphical Abstract. [file 7895293.f1.pdf]
